# Quality and reliability of myelodysplastic syndromes-related health information on Chinese short-video platforms: a multi-platform cross-sectional analysis

**DOI:** 10.3389/fdgth.2026.1956383

**Published:** 2026-09-09

**Authors:** Ruijun Yang, Wanjia Chen, Caixian Xu, Haotian Yan, Wenjie Dai, Yihui Lin, Zhimin Zhai, Wanqiu Zhang, Qianshan Tao

**Affiliations:** 1 Department of Hematology, The Second Affiliated Hospital of Anhui Medical University, Hefei City, China; 2 Anhui Medical University, Hefei, China

**Keywords:** digital health literacy, myelodysplastic syndromes, quality assessment, short-video platforms, social media platforms

## Abstract

**Background:**

Myelodysplastic syndromes (MDS) are common hematologic malignancies among older adults. With the rapid growth of short-video platforms, patients increasingly seek MDS-related health information online; however, the quality of MDS-related health information available on short-video platforms has not been systematically evaluated.

**Objective:**

This study assessed the quality and reliability of MDS-related short videos across three major Chinese platforms and examined the relationship between content quality and user engagement.

**Methods:**

Between June 25 and June 30, 2026, a total of 210 MDS-related videos were systematically collected from TikTok (Chinese version: Douyin), Bilibili, and Xiaohongshu (70 videos per platform). Video metadata were extracted, and content quality was independently evaluated using three validated instruments: the modified DISCERN (mDISCERN), the Journal of the American Medical Association (JAMA) benchmark criteria, and the Global Quality Scale (GQS). An overall quality score (SUM) was calculated by combining the three assessment tools. Spearman correlation analysis was performed to examine the associations between user engagement metrics and content quality.

**Results:**

Bilibili videos demonstrated the highest overall educational quality (mean SUM score: 9.79 ± 2.35), whereas TikTok generated the greatest user engagement (median number of likes: 81.00). Videos produced by professional creators achieved significantly higher scores across all quality assessment instruments than those produced by non-professional creators (mean SUM score: 8.81 vs. 5.03, *p* < 0.001). No significant associations were observed between any user engagement metric and content quality. Video duration was the only variable positively correlated with content quality (*r* = 0.332, *p* < 0.01).

**Conclusions:**

User engagement was disconnected from information quality in MDS-related short videos. Professional creators provided greater educational value without consistently receiving commensurate engagement. Digital health communication should prioritize expert-led content, transparent source attribution, and quality-informed dissemination.

## 1 Introduction

Myelodysplastic syndromes (MDS) are a heterogeneous group of clonal myeloid disorders originating from hematopoietic stem and progenitor cells, characterized by ineffective hematopoiesis, peripheral blood cytopenias, and a high risk of progression to acute myeloid leukemia (AML) (1–3). Clinically, MDS commonly presents with progressive anemia, recurrent infections, and bleeding tendencies, while some patients may also experience non-specific symptoms such as fatigue, palpitations, and reduced exercise tolerance (4, 5). MDS is recognized as one of the most prevalent bone marrow failure–related malignancies in the elderly population, with its incidence increasing significantly with age; consequently, the global burden of disease continues to rise in the context of population aging (3). Epidemiological studies indicate that MDS predominantly affects older individuals, with a median age at diagnosis exceeding 65 years, and a slightly higher incidence observed in males compared with females (6). In terms of disease progression, a subset of patients with MDS may evolve into AML during the disease course, particularly in high-risk subtypes, where the transformation risk is substantially elevated (7, 8). Although considerable advances have been achieved in recent years in risk stratification (e.g., IPSS-R and Molecular IPSS), supportive care, and disease-modifying therapies, including hypomethylating agents, immunomodulatory treatments, and allogeneic hematopoietic stem cell transplantation, the overall prognosis of MDS remains poor. Long-term survival is still limited, especially in elderly and high-risk patients (3, 9, 10).

Health education plays a crucial role in the management of myelodysplastic syndromes (MDS). It enables patients to acquire disease-related knowledge and develop the skills necessary for long-term self-management. Improving public awareness of MDS facilitates a better understanding of its pathogenesis, common clinical manifestations, and available treatment options, thereby promoting early recognition, timely intervention, and standardized management of the disease (11, 12). With the rapid advancement of information technology, the emergence of short-video platforms is reshaping traditional modes of health education dissemination (13). As an emerging form of social media, short videos, characterized by their intuitive, vivid, and convenient features, have gradually become an important channel for disseminating health-related knowledge (14, 15). Platforms such as TikTok, WeChat Channels, and Xiaohongshu have evolved beyond their original social and entertainment functions and are increasingly serving as important sources of health information and health education for the public (16).

With the rapid proliferation of short-video platforms, a substantial amount of content related to myelodysplastic syndromes (MDS) has emerged online. These videos cover a wide range of topics, including disease education, patient experience sharing, and discussions of various treatment strategies. However, the professional backgrounds and medical expertise of content creators vary considerably, resulting in substantial heterogeneity in video quality. This has also raised concerns regarding the accuracy, reliability, and educational value of such health-related content. Previous studies have demonstrated that many medical short videos lack sufficient scientific evidence, and some content creators may disseminate exaggerated or even unsubstantiated health information in pursuit of greater exposure, higher click-through rates, and increased user engagement (17, 18). For instance, certain videos may promote unverified self-management approaches, such as prolonged reliance on a single form of exercise, home remedies, or non-professional rehabilitation strategies (19). Such inappropriate recommendations may exacerbate patients' symptoms and potentially delay timely medical intervention (19, 20). Therefore, systematic and comprehensive evaluation of medical information on these platforms is essential to objectively assess content quality and credibility. This process not only contributes to improving the overall effectiveness of public health education but also ensures that patients and the general public have access to accurate and reliable medical information, thereby promoting evidence-based and rational disease management.

In recent years, researchers have frequently employed a variety of standardized assessment tools to comprehensively evaluate online health-related content from three key dimensions: information reliability, content completeness, and adherence to medical standards (21–24). For example, Yang et al. analyzed 176 short videos related to acute myeloid leukemia (AML) on TikTok and Bilibili, revealing that these videos primarily focused on treatment and prognosis-related information. Videos uploaded by hematologists achieved the highest quality scores; however, they tended to receive relatively lower levels of user engagement. In contrast, videos posted by patients, although attracting higher numbers of likes and comments, generally demonstrated poorer information quality, with incomplete content and, in some cases, unverified or potentially misleading claims regarding treatment and disease management strategies (25). Similar studies have also highlighted a consistent contradiction in this field, namely a clear disconnect between the “quality” and “popularity” of health-related content on short-video platforms (26–29).

To date, no study has systematically evaluated the quality of MDS-related health information on short-video platforms in China and abroad, despite MDS being a common hematologic malignancy in the elderly population with a substantial clinical and prognostic burden. This study critically evaluated the top-ranked MDS-related videos on Chinese short-video platforms, including TikTok, Bilibili, and Xiaohongshu. The assessment was conducted using three validated instruments—mDISCERN, the JAMA benchmark criteria, and the Global Quality Score (GQS)—to measure video quality, reliability, and educational value, respectively. This study, therefore, fills an important gap in the literature. In addition, this study compared differences in visibility and quality between videos uploaded by different types of content creators, including medical professionals and non-professional users. This comparison highlights both the role of short-video platforms in health education dissemination and their potential risks in spreading low-quality or unreliable medical information.

## 2 Materials and methods

### 2.1 Ethical considerations

This study analyzed only publicly available platform data, collected no identifiable user information or account IDs, and therefore required no ethical approval.

### 2.2 Video collection

Our search and data collection strategy followed established methodological guidelines for evaluating health-related content on online video platforms, which provide detailed procedures for healthcare professionals and researchers conducting digital health information studies. The search was performed between June 25 and June 30, 2026, across three major Chinese short-video platforms: TikTok, Bilibili, and Xiaohongshu. The three major platforms account for a substantial share of China's short-video market and differ markedly in both audience characteristics and content ecosystems. TikTok has a broad and diverse user base and is predominantly oriented toward general entertainment; Bilibili is characterized by a younger, knowledge-seeking audience and a strong knowledge-sharing community; whereas Xiaohongshu has a particularly strong presence in content related to women's health, wellness, and lifestyle. These differences in audience profiles and content ecosystems provide an important basis for comparative evaluation of health information quality across platforms. Videos were identified on each platform using the formal Chinese medical term “骨髓增生异常综合征” (myelodysplastic syndromes) as the search query, as this standardized nomenclature was considered most likely to retrieve clinically relevant educational content. To minimize potential bias introduced by personalized recommendation algorithms, searches were conducted in logged-out browser sessions (incognito/guest mode) without using any registered account, with the browser cache and browsing history cleared before each search. Searches were performed sequentially across platforms (TikTok, Bilibili, then Xiaohongshu) between 9:00 AM and 5:00 PM on each collection day to minimize diurnal variation. Videos were ranked according to the default recommendation algorithms of each platform, and no additional sorting or filtering criteria, such as relevance or view counts, were applied. Video metadata (likes, comments, shares, favorites, views, and duration) were recorded immediately at the time of search. This approach ensured that the collected videos reflected the standard ranking mechanisms of each platform.

The inclusion criteria were as follows: (1) publicly accessible videos; and (2) videos presented in Chinese. The exclusion criteria included: (1) videos uploaded within two weeks before the search date. This exclusion was applied because newly uploaded videos often undergo an algorithmic cold-start period during which content distribution and user interaction data (e.g.), likes, comments, shares, and favorites) may not yet reflect the video's actual visibility or audience reception. By requiring a minimum two-week interval since upload, all included videos had undergone comparable exposure periods and accumulated relatively stable engagement metrics, thereby minimizing temporal bias and ensuring comparability across platforms and videos; (2) commercial advertisements; and (3) duplicate or irrelevant videos; (4) repetitive reposts of identical content by the same or different uploaders, to avoid duplicate counting; (5) videos containing highly similar content derived from secondary editing or content repurposing. Potential duplicates or substantially similar videos were identified through manual review of video titles, thumbnails, and content descriptions. When multiple versions of substantially similar content were identified, only the earliest uploaded video was retained. The first 70 eligible videos from each platform were included for subsequent analysis. This sample size is consistent with established standards in multi-platform health information quality research, where studies typically include 50–100 videos per platform (30–33). This sampling captured the most visible content at collection rather than proportionally representing each platform's entire MDS video pool.

Extracted metadata included title, URL, likes, comments, shares, favorites, upload date, duration, days since upload, content focus, and creator type. Views were available only for Bilibili. Given this asymmetry, together with cross-platform differences in engagement mechanisms and data-reporting practices, engagement metrics were analyzed using the raw values publicly displayed on each platform, without cross-platform normalization. Accordingly, cross-platform comparisons reflect publicly observable engagement under each platform's native conditions rather than standardized engagement intensity adjusted for platform heterogeneity.

### 2.3 Video quality assessments

#### 2.3.1 Assessment instruments and scoring criteria

Video quality was evaluated with the JAMA benchmark criteria, Global Quality Score (GQS), and modified DISCERN (mDISCERN) (34–36). The SUM score was used as an exploratory composite index of complementary quality dimensions, calculated by summing the scores from the three assessment instruments (discern, JAMA, and GQS) to facilitate cross-platform comparisons and correlation analyses, consistent with previous social media health information studies (37). These tools assess structural and presentational quality, including authorship, purpose, sourcing, coherence, and uncertainty, rather than disease-specific accuracy or clinical appropriateness (38, 39). Detailed scoring criteria are provided in Supplementary Table S1 (22, 23, 40). Before consensus, inter-rater agreement for the GQS ratings, measured on an ordinal scale from 1 to 5, was assessed using unweighted Cohen's kappa coefficient.

To avoid ambiguity in terminology, the terms “video quality” and “content quality” were defined in this study as multidimensional constructs encompassing three key components: structural rigor, credibility of information sources, and reliability of the evidence presented. Structural rigor was assessed using the structure-related items of the Global Quality Score (GQS) and the modified DISCERN (mDISCERN) instrument. The credibility of information sources and the reliability of the information were evaluated using the JAMA benchmark criteria and the mDISCERN tool, whereas evidence-based presentation was reflected by the corresponding items within the mDISCERN assessment. It should be emphasized that these instruments evaluate the quality of information presentation rather than the scientific accuracy of the content itself. Therefore, the composite assessment scores reflect the overall educational value and information quality of each video, rather than its aesthetic production quality or the factual correctness of the medical information.

The JAMA Benchmark Criteria were used to assess the transparency and credibility of the information from four dimensions: authorship, attribution, disclosure, and currency. Each criterion was assigned a maximum of one point, yielding a total score ranging from 0 to 4. Scores of 0–1 indicated insufficient information, scores of 2–3 indicated partially sufficient information, and a score of 4 indicated fully sufficient information (40, 41). The GQS employs a 5-point Likert scale to evaluate the overall quality, flow, comprehensibility, and educational value of videos for the general audience. A score of 1 represents the lowest quality and limited educational value, whereas a score of 5 represents the highest quality and greatest educational usefulness modified DISCERN tool was used to evaluate the reliability and quality of health information for patients and healthcare providers (23, 42).

We used the five binary items in the first mDISCERN section to assess clarity of aims, relevance, balance, source transparency, and acknowledgment of uncertainty. Each “yes” scored one point (range 0–5), with higher scores indicating greater reliability.

#### 2.3.2 Classification of content creators

Two investigators with more than 10 years of hematology and oncology experience independently classified all 210 videos as professional or non-professional after standardized training; disagreements were resolved by consensus. Before consensus, inter-rater agreement for creator classification was high (94.8% agreement; 199/210 videos), indicating excellent agreement, and was quantified using Cohen's kappa coefficient and interpreted according to the Landis and Koch criteria: *κ* > 0.80 indicated excellent agreement, 0.60–0.80 substantial agreement, 0.40–0.60 moderate agreement, and <0.40 poor agreement (43).

Professional creators had publicly verifiable medical qualifications or healthcare/education affiliations and included: (1) licensed physicians, including hematologists, oncologists, general practitioners, and physicians from other specialties; (2) healthcare institutions, such as hospitals, clinics, health departments, and medical schools; (3) licensed non-physician healthcare professionals, including nurses, pharmacists, and medical technologists; and (4) health science communicators with formal medical training.

Non-professional creators lacked verifiable medical qualifications or institutional affiliations and included: (1) patients or caregivers sharing personal experiences with MDS; (2) general health or lifestyle content creators without disclosed medical backgrounds; and (3) unverified or anonymous creators whose professional backgrounds could not be determined.

Creator status was determined from profiles, verification badges, affiliations, and in-video credentials, with cross-checking against public institutional, licensing, or professional sources when possible. Ambiguous accounts were classified conservatively as non-professional. Initial agreement was 94.8% (199/210), and the remaining cases were resolved by consensus.

#### 2.3.3 Expert-based clinical concordance assessment of treatment information

A supplementary expert-based clinical concordance assessment was performed for the 129 videos (61.4%) that contained explicit treatment or disease-management recommendations. Two hematologists with more than 10 years of clinical experience in myelodysplastic syndromes independently assessed the videos. Each reviewer was blinded to the other reviewer's ratings and to the identities of the video creators. Treatment and management recommendations were evaluated for concordance with the Chinese Guidelines for Diagnosis and Treatment of Myelodysplastic Neoplasms (2026) (44). The assessment was conducted in June 2026 using the guideline version available at that time. Videos were classified according to their concordance with guideline-based recommendations into five predefined categories:

(1) Folk remedies/unverified supplements: presenting unsupported traditional formulations, supplements, or home remedies as primary or adjunctive MDS treatment.
(2) Delayed treatment: discouraging timely consultation, recommending prolonged observation without evaluation, or minimizing symptomatic disease.
(3) Under-treatment/over-treatment: recommending insufficient care for higher-risk disease or aggressive treatment for lower-risk asymptomatic patients.
(4) Incorrect medication discontinuation: advising unsupervised cessation or adjustment of prescribed therapies.
(5) Other inappropriate content: other clinically misleading or potentially harmful recommendations.

### 2.4 Statistical analysis

The statistical analysis in this study was conducted based on a dataset of all 210 included videos. Normality of distribution was assessed using the Shapiro–Wilk test, and homogeneity of variances was verified using Levene's test. Quality scores (mDISCERN, SUM, JAMA, and GQS) were approximately normally distributed with homogeneous variances across groups (all Levene's *p* > 0.05) and are presented as mean ± standard deviation (SD); between-platform comparisons for these variables were performed using one-way analysis of variance (ANOVA) with between-groups df = 2 and within-groups df = 207, followed by Tukey's honestly significant difference (HSD) *post hoc* test for pairwise comparisons. Effect sizes were reported as partial eta squared (*η*p^2^). Although these scores are bounded (e.g., mDISCERN 0–5, JAMA 0–4, GQS 1–5), ANOVA was deemed appropriate because the distributions did not deviate substantially from normality (all Shapiro–Wilk *p* > 0.05), variances were homogeneous across groups, and ANOVA is robust to moderate departures from normality with equal sample sizes across groups (*n* = 70 per platform). Non-normally distributed variables (likes, comments, shares, favorites, and video duration) are presented as median (interquartile range, IQR), and between-platform comparisons were performed using the Kruskal–Wallis H test, followed by Dunn's *post hoc* test with Bonferroni correction for multiple comparisons. Correlations between variables were assessed using Spearman's rank correlation coefficient. The internal consistency of the composite SUM score across the three component instruments was evaluated using Cronbach's alpha, with *α* ≥ 0.70 considered acceptable. Spearman's rank correlation was used to assess inter-scale correlations among the mDISCERN, JAMA, and GQS instruments, as well as associations between video characteristics, engagement metrics, and quality scores. Inter-rater reliability was evaluated using Cohen's kappa statistic. A two-tailed *P*-value of <0.05 was considered statistically significant. All statistical analyses were performed using IBM SPSS Statistics version 25, and visualizations were generated using GraphPad Prism version 9.

## 3 Results

### 3.1 Video retrieval and selection process

**Figure 1:**
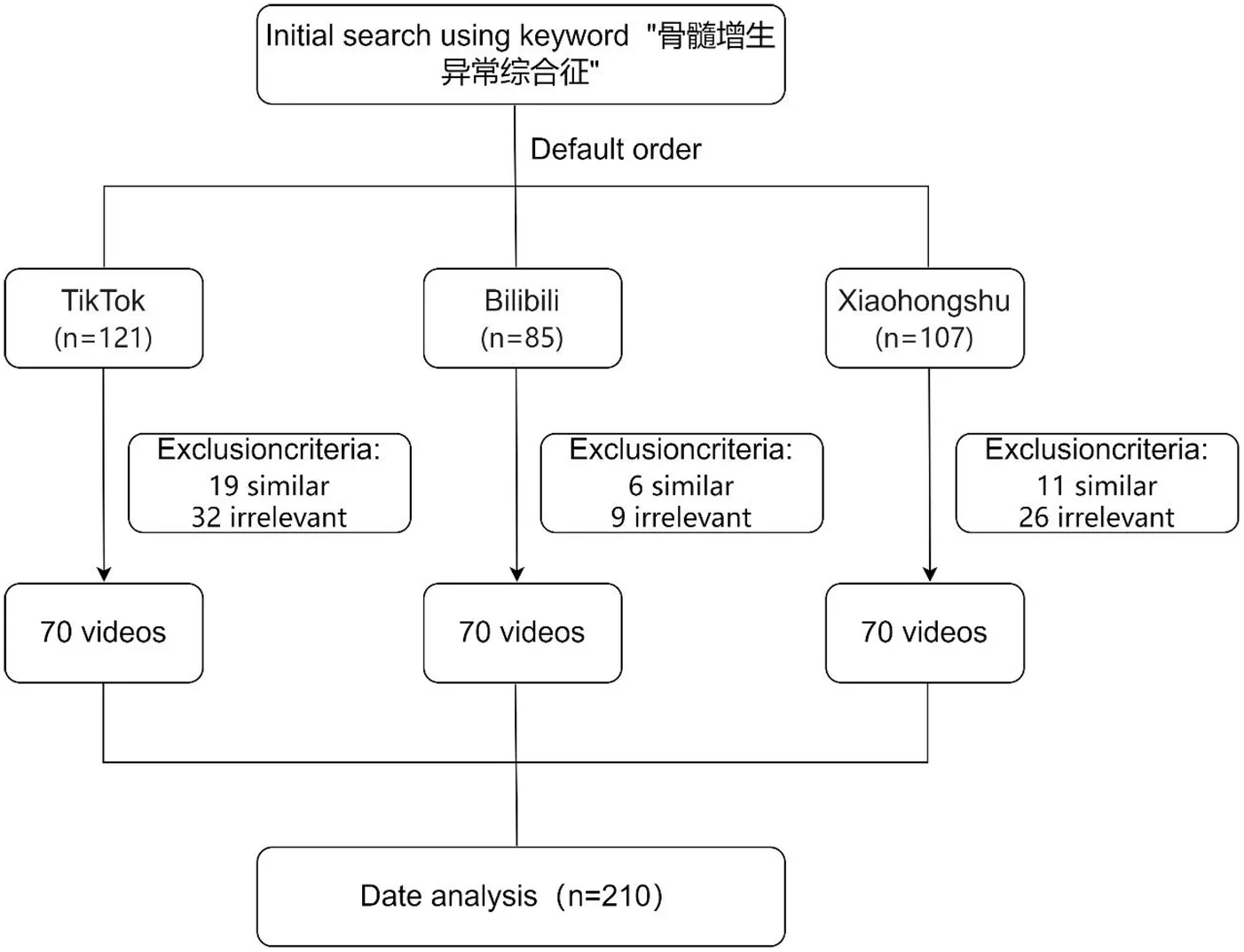
Flowchart of the study.

Of 313 screened videos, 70 per platform met eligibility criteria, yielding 210 videos for analysis with JAMA, GQS, and mDISCERN (Figure 1).

### 3.2 Characteristics of included videos

Table 1 summarizes video characteristics and engagement metrics.

**Table 1:** Comparison of video quality (mDISCERN, JAMA, and GQS scores) and user engagement metrics across TikTok, Bilibili, and Xiaohongshu platforms.

Variables	Total (*n* = 210)	Bilibili (*n* = 70)	TikTok (*n* = 70)	Xiaohongshu (*n* = 70)	Statistic	*P*
M Dis Score, Mean ± SD	2.78 ± 1.10	3.20 ± 0.99	2.67 ± 1.10	2.46 ± 1.10	*F* = 9.05	<0.001
SUM score, Mean ± SD	8.22 ± 2.62	9.79 ± 2.35	7.74 ± 2.22	7.13 ± 2.54	*F* = 23.99	<0.001
JAMA score, Mean ± SD	2.25 ± 0.90	2.77 ± 0.87	2.03 ± 0.70	1.94 ± 0.88	*F* = 21.48	<0.001
GQS score, Mean ± SD	3.20 ± 1.16	3.81 ± 1.08	3.04 ± 1.04	2.73 ± 1.10	*F* = 18.90	<0.001
Likes, M (Q1, Q3)	17.50 (4.00, 91.25)	27.50 (4.25, 121.00)	81.00 (34.75, 204.75)	4.00 (1.00, 11.50)	*χ*^2^ = 71.37^a^	<0.001
Comments, M (Q1, Q3)	1.00 (0.00, 7.75)	0.00 (0.00, 2.75)	9.00 (4.00, 42.25)	0.00 (0.00, 1.00)	χ^2^ = 87.94^a^	<0.001
Favorites, M (Q1, Q3)	10.50 (2.00, 59.75)	53.50 (4.00, 176.50)	18.00 (7.00, 99.00)	2.00 (0.00, 8.00)	χ^2^ = 51.76^a^	<0.001
Shares, M (Q1, Q3)	8.00 (1.00, 33.50)	12.50 (1.00, 45.75)	20.50 (4.00, 136.75)	2.00 (0.00, 6.75)	χ^2^ = 40.67^a^	<0.001
Duration, M (Q1, Q3)	95.00 (59.00, 242.75)	440.50 (133.00, 1,760.00)	62.00 (50.25, 98.50)	80.50 (50.75, 111.75)	χ^2^ = 72.58^a^	<0.001

F = one-way ANOVA F-statistic (df = 2, 207).

a Kruskal–Wallis H test χ^2^ statistic. *Post hoc* tests: Tukey HSD for ANOVA (quality scores), Dunn's test with Bonferroni correction for Kruskal–Wallis (engagement metrics). Levene's test confirmed homogeneity of variances for all quality scores (all *p* > 0.05). Effect sizes for ANOVA: partial eta squared (*η*p^2^) = 0.080 (mDISCERN), 0.188 (SUM), 0.172 (JAMA), 0.154 (GQS).

SD = standard deviation; M = median; Q1 = first quartile; Q3 = third quartile. Quality scores (mDISCERN, SUM, JAMA, GQS) are presented as mean ± SD. Engagement metrics (likes, comments, shares, favorites) and video duration are presented as median (Q1, Q3).

Bold values indicate statistically significant results (*p* < 0.05). *Post hoc* Tukey HSD tests: Bilibili scored significantly higher than both TikTok and Xiaohongshu across all quality instruments (all *p* ≤ 0.010); no significant differences between TikTok and Xiaohongshu (mDISCERN *p* = 0.459, SUM *p* = 0.279, JAMA *p* = 0.811, GQS *p* = 0.197). A two-tailed *p* < 0.05 was considered statistically significant. ns = not significant.

Comprehensive analysis using three assessment instruments and their composite SUM score revealed that videos on Bilibili demonstrated the highest overall quality. Specifically, Bilibili videos achieved significantly higher scores in mDISCERN (3.20 ± 0.99), SUM score (9.79 ± 2.35), JAMA benchmark criteria (2.77 ± 0.87), and GQS (3.81 ± 1.08) compared with the other two platforms (all *P* < 0.001, one-way ANOVA with Tukey HSD *post hoc* test; see Table 1 for detailed statistics).

In contrast, TikTok videos exhibited the highest publicly displayed raw engagement counts in this sample. The median number of likes (81.00), comments (9.00), and shares (20.50) on TikTok were significantly higher than those on Bilibili and Xiaohongshu (all *P* < 0.001, Kruskal–Wallis H test with Dunn's *post hoc* Bonferroni correction). Notably, Bilibili videos had the highest median number of saves (53.50) and substantially longer durations, with a median length of 440.50 s. In comparison, videos on TikTok and Xiaohongshu were considerably shorter, with median durations of 62.00 s and 80.50 s, respectively.

### 3.3 Characteristics of content uploaders

**Figure 2:**
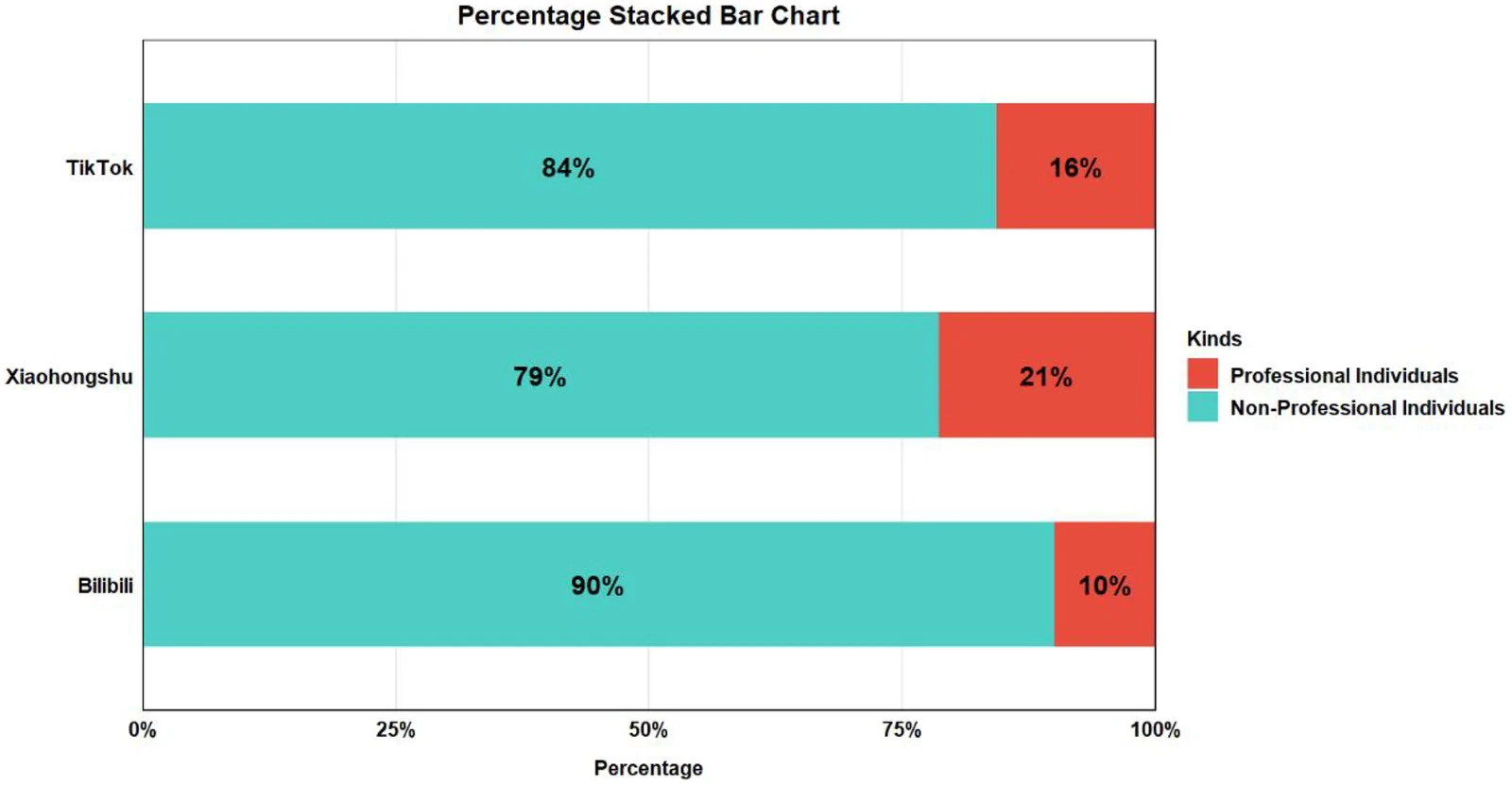
Distribution patterns of professional vs. non-professional content creators across platforms.

Professional creators produced 90% of Bilibili, 84% of TikTok, and 79% of Xiaohongshu videos (Figure 2). These differences may reflect platform ecology: Bilibili's knowledge-sharing community may attract more professional creators, whereas Xiaohongshu's lifestyle orientation encourages user-generated content.

To further evaluate the influence of uploader background on video quality and user engagement, we compared videos produced by professional and non-professional creators. Professional creators (*n* = 177) achieved significantly higher scores across all quality assessment instruments than non-professional creators (*n* = 33) (all *p* < 0.001, independent samples *t*-tests; see Table 2 for detailed statistics). The mean mDISCERN score was 2.99 ± 0.98 vs. 1.64 ± 1.03. The mean SUM score was 8.81 ± 2.23 vs. 5.03 ± 2.27. The mean JAMA score was 2.36 ± 0.89 vs. 1.64 ± 0.70, and the mean GQS score was 3.46 ± 0.97 vs. 1.76 ± 1.06. All differences were statistically significant (all *p* < 0.001). Regarding user engagement, videos uploaded by professional creators received significantly more likes (median: 32.00 vs. 8.00; *p* = 0.016) and favorites (median: 14.00 vs. 7.00; *p* = 0.021). They also had a significantly longer duration (99.00 vs. 71.00 s; *p* = 0.024). In contrast, no significant differences were observed in the numbers of comments (*p* = 0.406) or shares (*p* = 0.144) between the two groups (Table 2). These findings suggest that professional creators are more likely to provide higher-quality health information and achieve better audience engagement on selected metrics.

**Table 2:** Cross-platform comparison of video quality and engagement metrics between professional and non-professional creators.

Variables	Total (*n* = 210)	Non-professional creators (*n* = 33)	Professional creators (*n* = 177)	Statistic	*P*
M Dis Score, Mean ± SD	2.78 ± 1.10	1.64 ± 1.03	2.99 ± 0.98	*t* = −7.00	<0.001
SUM score, Mean ± SD	8.22 ± 2.62	5.03 ± 2.27	8.81 ± 2.23	*t* = −8.81	<0.001
JAMA score, Mean ± SD	2.25 ± 0.90	1.64 ± 0.70	2.36 ± 0.89	*t* = −5.23	<0.001
GQS score, Mean ± SD	3.20 ± 1.16	1.76 ± 1.06	3.46 ± 0.97	*t* = −8.58	<0.001
Likes, M (Q1, Q3)	17.50 (4.00, 91.25)	8.00 (3.00, 18.00)	32.00 (5.00, 108.00)	*Z* = −2.42	0.016
Comments, M (Q1, Q3)	1.00 (0.00, 7.75)	1.00 (0.00, 5.00)	2.00 (0.00, 8.00)	*Z* = −0.80	0.406
Favorites, M (Q1, Q3)	10.50 (2.00, 59.75)	7.00 (1.00, 17.00)	14.00 (2.00, 74.00)	*Z* = −2.30	0.021
Shares, M (Q1, Q3)	8.00 (1.00, 33.50)	4.00 (1.00, 16.00)	9.00 (1.00, 36.00)	*Z* = −1.46	0.144
Duration, M (Q1, Q3)	95.00 (59.00, 242.75)	71.00 (57.00, 111.00)	99.00 (60.00, 293.00)	*Z* = −2.25	0.024

t = independent samples *t*-test (df = 208); *Z* = Mann–Whitney *U*-test statistic. Effect sizes for *t*-tests: Cohen's d = 1.367 (mDISCERN), 1.690 (SUM), 0.841 (JAMA), 1.731 (GQS).

SD = standard deviation; M = median; Q1 = first quartile; Q3 = third quartile. Quality scores are presented as mean ± SD and compared using independent samples *t*-tests. Engagement metrics and video duration are presented as median (Q1, Q3) and compared using Mann–Whitney *U*-tests.

Bold values indicate statistically significant results (*p* < 0.05). Professional creators achieved significantly higher quality scores than non-professional creators across all instruments (all *p* < 0.001). A two-tailed *p* < 0.05 was considered statistically significant.

### 3.4 Video quality and reliability assessment

#### 3.4.1 Inter-rater agreement

Two trained medical reviewers independently assessed all videos. Agreement for the GQS score was excellent (weighted Cohen's *κ* = 0.929), indicating almost perfect agreement by Landis–Koch criteria.

The confusion matrix (Table 3) further demonstrated the robustness of the scoring process. Among videos assigned a score of 3, 84 of 87 were rated identically by both reviewers. Similarly, agreement was observed for 33 of 34 videos assigned a score of 5. The few discrepancies were primarily confined to adjacent rating categories (e.g., scores of 3 vs. 4 and 4 vs. 5), with no substantial disagreements across non-adjacent categories. These results indicate excellent inter-rater reliability and support the robustness of the video quality assessment process.

**Table 3:** Inter-rater agreement in video quality assessment.

Cohen's Kappa coefficient = 0.929						
Coder A	Coder B					SUM
	3	4	5	2	1	
3	84	1	0	2	0	87
4	2	39	1	0	0	42
5	0	1	33	0	0	34
2	2	0	0	22	1	25
1	0	0	0	0	21	22
SUM	88	41	34	25	22	210

#### 3.4.2 Video quality across platforms

As shown in Supplementary Figure S1, the SUM score had acceptable internal consistency (Cronbach's *α* = 0.764), indicating related but nonredundant quality dimensions; note that this exploratory composite index does not imply a validated unidimensional scale. Inter-scale Spearman correlations were moderate and significant: mDISCERN-JAMA (*r* = 0.487), mDISCERN-GQS (*r* = 0.575), and JAMA-GQS (*r* = 0.502) (all *p* < 0.001), indicating related but nonredundant quality dimensions, supporting convergence while preserving the multidimensional nature of the quality construct.

**Figure 3:**
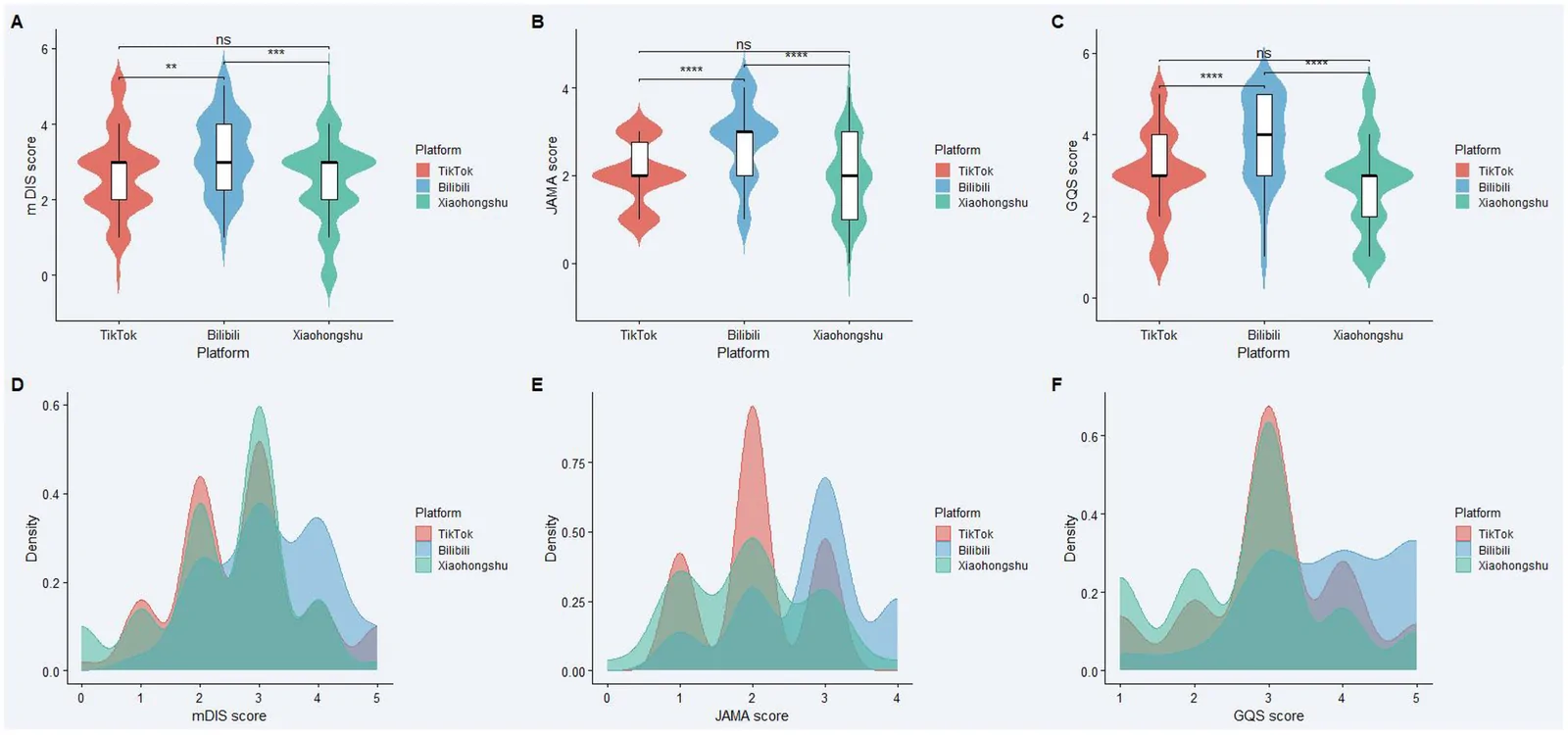
Cross-platform comparison of video quality metrics and subdomain scores. **(A–C)** Violin plots showing the distribution of mDISCERN, JAMA, and GQS scores across TikTok, Bilibili, and Xiaohongshu. Statistical comparisons were performed using one-way ANOVA followed by Tukey HSD *post hoc* test. **(D–F)** Ridge density plots for each scoring tool, illustrating platform-specific score concentration and heterogeneity. *** indicates *p* < 0.001; **** indicates *p* < 0.0001.

Figures 3A–C shows that Bilibili had the highest median scores on all three instruments, followed by TikTok and Xiaohongshu.

Statistical comparisons confirmed significant between-platform differences (one-way ANOVA for quality scores; Kruskal–Wallis H test for engagement metrics and video duration). Bilibili had higher mean mDISCERN (3.20 ± 0.99), SUM (9.79 ± 2.35), JAMA (2.77 ± 0.87), and GQS (3.81 ± 1.08) scores than TikTok (2.67 ± 1.10, 7.74 ± 2.22, 2.03 ± 0.70, and 3.04 ± 1.04, respectively) and Xiaohongshu (2.46 ± 1.10, 7.13 ± 2.54, 1.94 ± 0.88, and 2.73 ± 1.10, respectively). Tukey's HSD tests showed that Bilibili scored significantly higher than both TikTok and Xiaohongshu across all four quality measures (all adjusted *P* ≤ 0.010), whereas no significant differences were observed between TikTok and Xiaohongshu for mDISCERN (*P* = 0.459), SUM (*P* = 0.279), JAMA (*P* = 0.811), or GQS (*P* = 0.197). Overall, the included Bilibili videos received consistently higher assessed quality scores than those from the other two platforms.

To further characterize score concentration and distributional patterns, density plots were constructed (Figures 3D–F), corroborating the findings observed in the violin plots. For mDISCERN, Bilibili exhibited a distribution skewed toward higher scores, with a peak around 3, reflecting generally higher content quality. In contrast, TikTok and Xiaohongshu showed broader distributions concentrated between 1 and 3, with a greater proportion of low scores. JAMA score distributions followed a consistent pattern: Bilibili scores clustered between 2 and 3, indicating stronger compliance with authorship, attribution, currency, and disclosure standards, whereas TikTok and Xiaohongshu scores concentrated between 1 and 2, suggesting comparatively poorer adherence. GQS density plots similarly demonstrated that Bilibili scores were concentrated at higher values, while TikTok and Xiaohongshu displayed wider, lower-scoring distributions.

**Figure 4:**
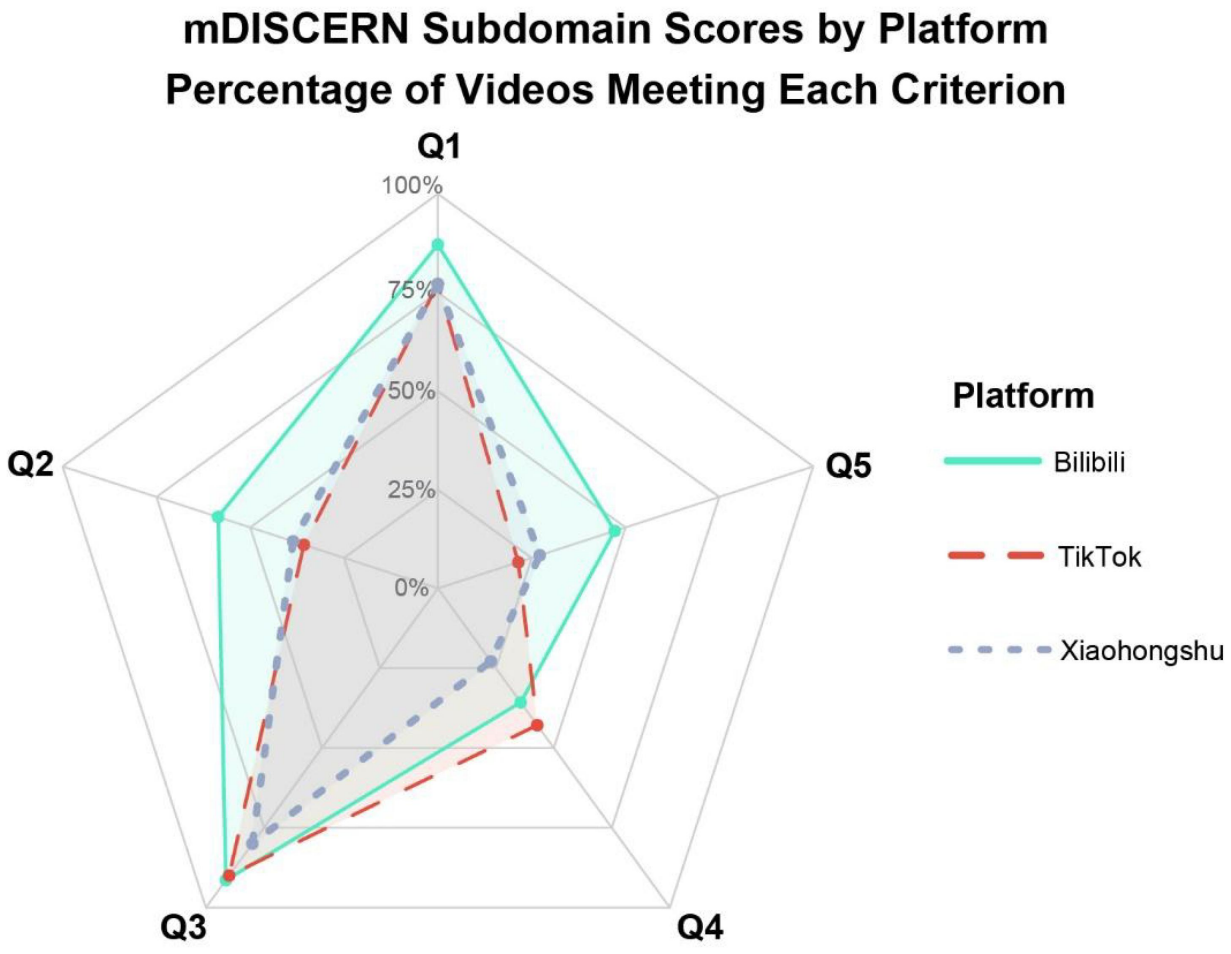
Radar chart showing the mDISCERN subdomain scores (Q1–Q5) across three platforms.

To further explore the contribution of individual quality domains to inter-platform differences, the five mDISCERN subdomains (Q1–Q5) were analyzed separately (Figure 4). Regarding clarity of aims (Q1), the three platforms showed comparable performance, with Bilibili (87.1%) scoring slightly higher than both TikTok (77.1%) and Xiaohongshu (77.1%). For source citation (Q2), Bilibili (58.6%) outperformed TikTok (35.7%) and Xiaohongshu (38.6%), although scores remained generally low across all platforms. Relevance (Q3) achieved the highest scores among all domains, reaching 91.4% for Bilibili, 90.0% for TikTok, and 80.0% for Xiaohongshu, with relatively small differences between platforms. In terms of information reliability (Q4), TikTok (42.9%) scored slightly higher than Bilibili (35.7%) and Xiaohongshu (22.9%); however, the overall performance remained suboptimal. Discussion of uncertainty (Q5) received the lowest scores across all platforms, with Bilibili (47.1%) performing better than TikTok (21.4%) and Xiaohongshu (27.1%).

Overall, the radar plot showed that Bilibili exhibited a larger and more balanced profile, indicating superior performance across multiple quality domains, particularly in source citation and discussion of uncertainty. In contrast, TikTok and Xiaohongshu displayed smaller and less balanced profiles, with notable deficiencies in Q2 and Q5. These findings suggest that videos on these two platforms frequently lack adequate citation of reliable sources and rarely acknowledge the limitations or uncertainties of the information presented, reflecting a tendency toward fragmented content and reduced information quality.

### 3.5 Correlation analysis

To examine the relationship between user engagement and video quality, Spearman rank correlation analysis was conducted on six key variables: Likes, Comments, Shares, Favorites, video duration (in seconds), and a composite SUM quality score (aggregated from mDISCERN, JAMA, and GQS instruments).

As shown in Table 4, the four core engagement metrics—Likes, Comments, Shares, and Favorites—demonstrated moderate-to-high intercorrelations, all statistically significant at *p* < 0.01. Likes were correlated with Comments (*r* = 0.767), Shares (*r* = 0.862), and Favorites (*r* = 0.877), indicating notable multicollinearity among these variables. This pattern indicates that engagement metrics tended to co-occur across videos, and that higher engagement metrics were not associated with higher intrinsic content quality. In contrast, Video Duration showed no significant association with Likes (*r* = −0.016, *p* = 0.823), Comments (*r* = −0.012, *p* = 0.859), Shares (*r* = −0.017, *p* = 0.807), or Favorites (*r* = 0.051, *p* = 0.458). Overall, engagement metrics showed moderate-to-high intercorrelations (r range: 0.588–0.903), with shares and favorites being the most strongly paired (*r* = 0.903).

**Table 4:** Spearman rank correlation matrix for engagement metrics, video duration, and composite quality score (*N* = 210).

Variables	Correlation	Likes	Comments	Shares	Favorites	Duration	SUM score
Likes	Spearman Correlation	1	0.767**	0.862**	0.877**	−0.016	0.037
	Sig. (2−tailed)		0	0	0	0.823	0.598
	Number of Cases	210	210	210	210	210	210
Comments	Spearman Correlation	0.767**	1	0.643**	0.588**	−0.012	0.025
	Sig. (2−tailed)	0		0	0	0.859	0.714
	Number of Cases	210	210	210	210	210	210
Shares	Spearman Correlation	0.862**	0.643**	1	0.903**	−0.017	0.022
	Sig. (2−tailed)	0	0		0	0.807	0.754
	Number of Cases	210	210	210	210	210	210
Favorites	Spearman Correlation	0.877**	0.588**	0.903**	1	0.051	0.073
	Sig. (2−tailed)	0	0	0		0.458	0.294
	Number of Cases	210	210	210	210	210	210
Duration	Spearman Correlation	−0.016	−0.012	−0.017	0.051	1	0.332**
	Sig. (2−tailed)	0.823	0.859	0.807	0.458		0
	Number of Cases	210	210	210	210	210	210
SUM score	Spearman Correlation	0.037	0.025	0.022	0.073	0.332**	1
	Sig. (2−tailed)	0.598	0.714	0.754	0.294	0	
	Number of Cases	210	210	210	210	210	210

Values are presented as Spearman rank correlation coefficients (r), with corresponding two-tailed *P* values.

** *P* < 0.01. *P* values displayed as 0 indicate *P* < 0.001 after rounding. SUM represents the composite quality score calculated from the mDISCERN, JAMA, and GQS scores.

SUM was not significantly correlated with likes (*r* = 0.037, *p* = 0.598), comments (*r* = 0.025, *p* = 0.714), shares (*r* = 0.022, *p* = 0.754), or favorites (*r* = 0.073, *p* = 0.294), indicating that popularity did not reflect educational value.

Duration was moderately correlated with SUM (*r* = 0.332, *p* < 0.01). Longer video duration was positively associated with the composite quality score; however, this unadjusted correlation does not establish that increasing video duration improves content quality.

Engagement and educational quality were therefore disconnected. High interaction may reflect popularity or entertainment appeal but should not be treated as an indicator of health-information quality.

### 3.6 Expert-based clinical concordance of treatment information and prevalence of potentially inappropriate content

Among the 210 included videos, 129 (61.4%) contained explicit treatment recommendations or management advice and were subject to clinical accuracy assessment. Treatment categorization was performed independently by two senior hematologists, with discrepancies resolved through consensus discussion. Overall, 106 videos (82.2%) provided correct treatment information aligned with current evidence-based guidelines, whereas 23 videos (17.8%) contained inappropriate content with potential for clinical harm.

**Figure 5:**
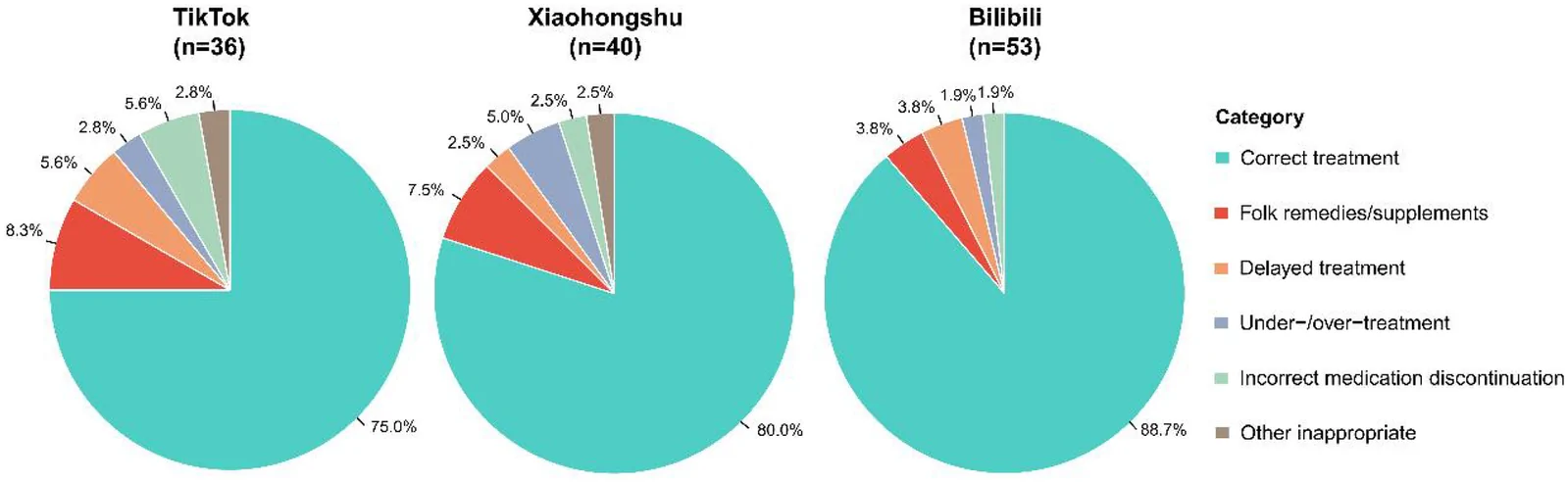
Treatment information accuracy across three short-video platforms.

Cross-platform comparison revealed differences in treatment accuracy, though these did not reach statistical significance (*χ*^2^ = 2.92, *p* = 0.232) (Table 5; Figure 5). Bilibili demonstrated the highest proportion of correct content (88.7%, 47/53), followed by Xiaohongshu (80.0%, 32/40) and TikTok (75.0%, 27/36). While the difference was not statistically significant, likely due to the relatively modest sample sizes within the treatment-related video subset, the trend favored Bilibili's superior content accuracy, consistent with the platform's higher structural quality scores reported in Section 3.4.

**Table 5:** Treatment information accuracy by platform and creator type.

Variable	*n*	Correct treatment *n* (%)	Inappropriate treatment *n* (%)	χ^2^	*P*-value
Overall	129	106 (82.2)	23 (17.8)	—	—
Platform				2.92	0.232
TikTok	36	27 (75.0)	9 (25.0)		
Xiaohongshu	40	32 (80.0)	8 (20.0)		
Bilibili	53	47 (88.7)	6 (11.3)		
Creator type				28.83	<0.001
Professional creators	103	94 (91.3)	9 (8.7)		
Non-professional creators	26	12 (46.2)	14 (53.8)		

Data are presented as *n* (%). χ^2^ tests were used to compare the proportions of correct vs. inappropriate treatment content across platforms and between creator types. “—” indicates that the statistic was not applicable for the overall row. Treatment-related videos were defined as those containing explicit treatment recommendations or management guidance (*n* = 129 of 210 total videos).

Stratified analysis by creator type revealed a stark and statistically significant difference (*χ*^2^ = 28.83, *p* < 0.001). Professional creators produced substantially more accurate treatment content (91.3%, 94/103) compared with non-professional creators (46.2%, 12/26). Notably, among the 23 videos containing inappropriate content, 14 (60.9%) were uploaded by non-professional creators, despite this group accounting for only 20.2% (26/129) of treatment-related videos. This finding underscores the critical role of medical expertise and professional training in ensuring the clinical appropriateness of health information disseminated through short-video platforms.

## 4 Discussion

This first multi-platform evaluation of MDS-related short videos found moderate-to-low quality, substantial platform differences, and little correspondence between engagement and educational value.

Bilibili consistently scored higher than TikTok and Xiaohongshu on every quality measure, an advantage that likely stems from its platform ecosystem. Because Bilibili positions itself around knowledge sharing and educational content, it tends to draw in a larger share of healthcare professionals and medical institutions, which in turn elevates the quality of health-related material available on the platform (45). Videos hosted on Bilibili were also considerably longer than those on the other two platforms. TikTok and Xiaohongshu, by contrast, are oriented more toward entertainment and lifestyle content, which lowers the barrier for non-professional creators to participate. This compositional difference among creators may go some way toward explaining the comparatively lower content quality observed on these platforms (16). These results align with earlier cross-platform work by Xu et al. and Xia et al., both of whom found that Bilibili offered significantly higher-quality health information relative to other mainstream Chinese social media (46, 47). That said, even Bilibili reached only a partially sufficient quality threshold, pointing to substantial room for improvement in how MDS-related health information is standardized and verified.

Before interpreting these between-platform differences, the measurement properties of the composite assessment warrant consideration. The acceptable internal consistency of the SUM score and moderate correlations among mDISCERN, JAMA, and GQS suggest that these instruments assess related but nonredundant aspects of video quality. Rather than necessarily indicating poor measurement, this pattern is consistent with the multidimensional nature of health information quality, as the three instruments emphasize information reliability, source transparency, and educational utility, respectively (48, 49). The SUM score should therefore be interpreted as an integrated summary of complementary quality dimensions rather than as a strictly unidimensional measure.

When the mDISCERN subdomains were analyzed individually, source attribution (Q2) and discussion of uncertainty (Q5) emerged as the weakest dimensions on all three platforms, while relevance of information (Q3) scored highest. This pattern indicates that creators generally kept their content focused on MDS, yet seldom cited credible sources or acknowledged the limitations inherent in current medical evidence. Because MDS involves complex prognostic stratification and highly individualized treatment approaches (50), the lack of transparent sourcing combined with insufficient discussion of uncertainty could distort patients' understanding of their condition and, by extension, shape their healthcare-seeking behavior and treatment adherence in problematic ways (51, 52). Zheng et al. documented a similar phenomenon among lung cancer–related short videos, underscoring that transparent citation practices are central to the perceived credibility of online medical content (27).

Professional creator status was associated with higher assessed video quality. Content produced by professional creators scored significantly higher on all quality instruments compared with that of non-professional creators, and also accumulated more likes and favorites. This observation pushes back against the widely held assumption that professionally made medical content is inherently less appealing to audiences. Yang et al. previously reported that hematologist-produced videos achieved the highest information quality among AML-related content but attracted relatively limited engagement (25). Our results tell a somewhat different story: professional creators outperformed their non-professional counterparts not only in content quality but also in selected engagement metrics, which may signal growing public receptiveness to rigorous medical information. It is worth noting, however, that comment and share counts did not differ significantly between the two groups, implying that these particular engagement behaviors are shaped more by emotional resonance or topic appeal than by informational rigor (17, 53).

Beyond these differences in overall information quality, our clinical accuracy assessment identified potentially harmful information in 23 of the 129 treatment-related videos (17.8%), raising concerns about the potential scale of exposure on social media. Professional creators demonstrated significantly higher treatment accuracy than non-professional creators (91.3% vs. 46.2%; *P* < 0.001); nevertheless, 8.7% of professionally produced videos still contained inappropriate treatment information, possibly owing to insufficient familiarity with evolving MDS guidelines or the oversimplification of complex clinical concepts. This issue is particularly concerning because MDS predominantly affects older adults, who may have limited digital health literacy and greater difficulty distinguishing evidence-based recommendations from unverified claims (54–56). These findings reinforce the need for stronger professional oversight, platform-based moderation of inappropriate or potentially harmful health information, and clinician-led guidance to help patients critically evaluate online health information.

The correlation analysis produced what is arguably the central finding of this study: content quality scores bore no significant association with any user engagement metric. By contrast, likes, comments, shares, and favorites correlated moderately to strongly with each other, suggesting that engagement metrics tended to co-occur, potentially reflecting shared visibility, audience preferences, or other platform-specific factors (57). TikTok illustrates this disconnect vividly—it generated far greater user engagement than either Bilibili or Xiaohongshu despite registering the comparatively lower content quality overall. Previous studies have reported a similar mismatch between popularity and informational value (58, 59). The weak correlation between content quality and user engagement observed in this study may have multiple explanations. One possible explanation is that platform recommendation algorithms, which are generally designed to maximize user engagement and session duration (60, 61), may favor content that elicits rapid emotional or affective responses, potentially giving entertaining or emotionally engaging content greater visibility than more comprehensive educational content (57, 62). However, because this cross-sectional study did not directly measure recommendation pathways, exposure rates, impressions, or algorithmic distribution mechanisms, this interpretation remains a plausible hypothesis rather than a confirmed finding.

Duration was the only variable positively associated with quality, consistent with the possibility that longer videos allow greater explanation and sourcing (63, 64). However, Bilibili had both the longest videos and highest scores, and professional creators produced longer, higher-quality videos; platform and creator type may therefore confound this association. Multivariable analyses are needed to establish an independent duration effect. Taken together, these findings suggest that elevating the visibility of high-quality medical content is unlikely to happen through market dynamics alone; deliberate platform-level intervention will be necessary.

These results carry practical implications for how MDS-related health information is disseminated via short-video platforms. On the platform side, exploring mechanisms to integrate creator credential verification into content recommendation systems and increase the visibility of content from verified healthcare professionals would facilitate broader circulation of quality-assured medical material. On the creator side, incentive structures—continuing medical education credits, institutional endorsements, and similar mechanisms—could motivate more clinicians to engage in short-video health communication, with particular attention to evidence-based content that cites sources transparently and runs long enough to convey complex medical ideas adequately (39). On the user side, strengthening digital health literacy and cultivating the capacity to critically appraise online medical information remain essential for mitigating the influence of low-quality or unreliable health information (65). These considerations carry special weight for patients with MDS, a population that skews older, bears a considerable disease burden, and has pronounced informational needs; accurate, well-standardized educational content can directly support their decision-making and disease management (66). These measures are particularly critical for MDS patients, who are predominantly elderly (median age >65 years) and may possess lower levels of digital health literacy, rendering them more vulnerable to unreliable or unsubstantiated health information and less equipped to critically appraise online health content. Moving forward, tighter collaboration among clinicians, platform developers, and public health policymakers will be critical to bringing the online health information landscape into closer alignment with evidence-based principles.

## 5 Conclusion

This cross-platform analysis reveals a clear disconnect between educational quality and user engagement for MDS-related short videos on Chinese social media platforms. The social media data was accessed short videos on Chinese social media platforms. Content produced by healthcare professionals consistently demonstrated superior informational value but did not receive proportionally higher user interaction, suggesting that current visibility patterns on these platforms may not effectively direct audiences toward the most reliable health information. Professional creators were well-represented among top-ranked search results, indicating that authoritative content is discoverable through direct search. However, content quality showed weak or selective associations with engagement metrics and should not be inferred from popularity alone; once accessed, high-quality content does not consistently generate more user interaction than lower-quality material. Clinically inappropriate information was identified in 17.8% of treatment-related videos, although treatment accuracy was significantly higher among professional than non-professional creators. Such potentially inappropriate treatment information may pose substantial risks to clinical decision-making among older patients with MDS, underscoring the need for stronger professional oversight and content moderation. Addressing this disconnect will require multi-stakeholder efforts, including expert credential verification, transparent source attribution, and enhanced public digital health literacy to empower patients and caregivers to critically evaluate online health information and make evidence-based decisions in the management of MDS.

### 5.1 Limitations

This study has several limitations. First, its cross-sectional design and inclusion of only three Chinese platforms limit the temporal and geographic generalizability of the findings. The relatively small sample size further restricts external validity; future studies should include larger samples and longitudinal monitoring. Moreover, the equal inclusion of 70 top-ranked videos per platform represented different proportions of the total MDS-related content available on each platform and may therefore not reflect platform-wide quality distributions. The sample also provides only a snapshot of content available at a single time point rather than a comprehensive inventory. Accordingly, these findings apply only to the sampled top-ranked MDS-related content on the three selected Chinese short-video platforms during the study period and should not be generalized to other diseases, platforms, countries, or time points. Although inter-rater agreement was high (Cohen's *κ* = 0.929), some subjectivity in quality assessment was unavoidable (42). In addition, the instruments primarily evaluate the structural and presentational quality of information and cannot determine whether the content is consistent with clinical guidelines or contains potentially harmful medical inaccuracies; disease-specific accuracy assessment tools are therefore needed. Because the publicly disclosed credentials of all creators could not be independently verified, some misclassification may have occurred despite a classification agreement of 94.8% and the conservative handling of ambiguous cases. View-count data were available only for Bilibili, and differences in interaction mechanisms and data-reporting practices across platforms mean that comparisons of raw engagement metrics reflect publicly displayed performance under each platform's native conditions rather than standardized levels of user activity. Without multivariable or platform-stratified analyses, whether video duration was independently associated with content quality could not be determined. Using only the formal medical term as the search query may have under-sampled colloquial, patient-oriented content while overrepresenting professional medical sources, thereby limiting the generalizability of the findings. Finally, recommendation mechanisms and algorithm-driven exposure were not directly measured; related interpretations therefore remain hypotheses based on correlational evidence and require validation using platform-level data or experimental studies.

## Data Availability

The raw data supporting the conclusions of this article will be made available by the authors, without undue reservation.
