# Supplementary material for: Quality and reliability of myelodysplastic syndromes-related health information on Chinese short-video platforms: a multi-platform cross-sectional analysis
Source: Front Digit Health. 2026 Sep 9;8:1956383. doi: 10.3389/fdgth.2026.1956383 (PMC13597896; doi:10.3389/fdgth.2026.1956383)
Supplement: Supplementary file 1 [file Image1.pdf]

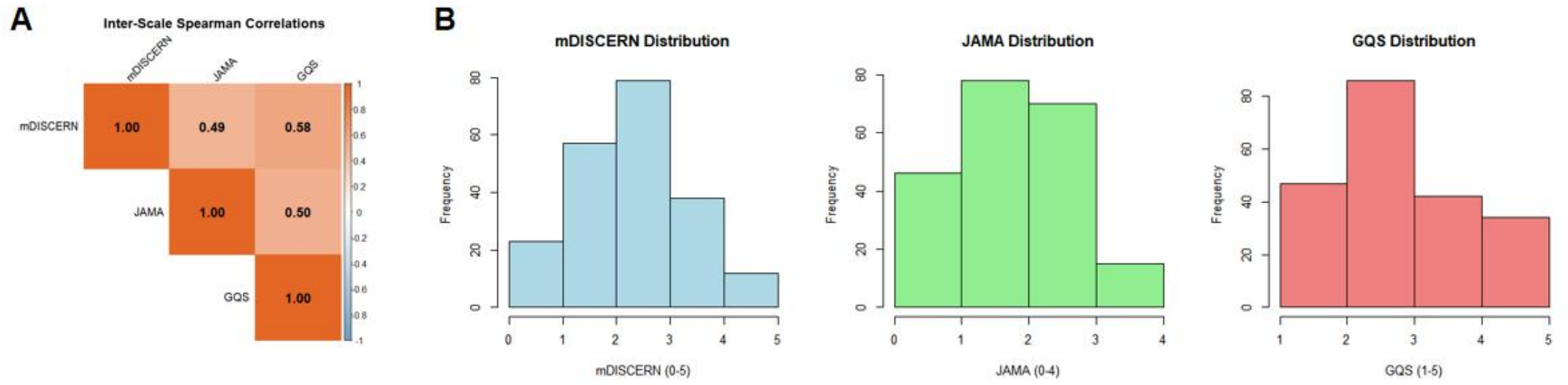

Figure S1. Internal Consistency and Distribution Characteristics of Quality Assessment Instruments.

(A) Inter-scale Spearman correlations among mDISCERN (0-5), JAMA (0-4), and GQS (1-5). Color intensity represents correlation strength. Correlation coefficients: mDISCERN-JAMA ($r = 0.49$), mDISCERN-GQS ($r = 0.58$), JAMA-GQS ($r = 0.50$), all $p < 0.001$. Moderate positive correlations indicate instruments assess related but partially distinct quality dimensions, supporting their combination (Cronbach's $\alpha = 0.764$). (B) Score distributions for mDISCERN (blue), JAMA (green), and GQS (coral). mDISCERN approximately normal (mode = 3), JAMA positively skewed (mode = 1-2), GQS near-uniform (mode = 2). Distinct distributional patterns support complementary measurement of quality dimensions. n = 210 videos.
