# Supplementary material for: Quality and reliability of myelodysplastic syndromes-related health information on Chinese short-video platforms: a multi-platform cross-sectional analysis
Source: Front Digit Health. 2026 Sep 9;8:1956383. doi: 10.3389/fdgth.2026.1956383 (PMC13597896; doi:10.3389/fdgth.2026.1956383)
Supplement: Supplementary file 2 [file Table1.pdf]

Supplementary: Global Quality Score (GQS) (Scoring ranges from 1 to 5)

GQS Definition	Score
Poor quality：Specifically, the content is illogical, the mobility is poor, most of the information is missing, and it is useless for patients.	1
Generally poor quality ：the content logic is poor, although some information is listed, more important information is still missing, and the use of patients is very limited.	2
Moderate quality：some important information is adequately discussed.	3
Good quality and flow：Specifically, the video logic is clear and smooth, covering most of the relevant information, which is useful for patients.	4
Excellent quality and flow：Specifically, the video logic is clear, and the content is very smooth, which is very useful for patients.	5

The Journal of the American Medical Association (JAMA) benchmark criteria.

Score*	Score component	
1 score	Authorship	Author and contributor credentials and their affiliations should be provided.
1 score	Attribution	Clearly lists all copyright information and states references and sources for content.
1 score	Currency	Initial date of posted content and subsequent updates to content should be provided.
1 score	Disclosure	Conflicts of interest, funding, sponsorship, advertising, support, and video ownership should be fully disclosed.

*The criteria of each aspect were scored separately, and 1 point for each criterion with a total score of 4 points.

Supplementary: Modified DISCERN quality criteria for assessing the reliability of video. (1 point for answer ‘yes’, 0 point for answer ‘no’)

**Reliability Score**
1. Is the video clear, concise, and understandable?
2. Are valid sources cited?
3. Is the content presented balanced and unbiased?
4. Are additional sources of content listed for patient reference?
5. Are areas of uncertainty mentioned?
